# Editorial: Antioxidants in Autism Spectrum Disorders

**DOI:** 10.3389/fpsyt.2022.889865

**Published:** 2022-04-07

**Authors:** Francesca Bonomini, Dario Siniscalco, Stephen Schultz, Carla Carnovale, Catherine Barthélémy, Elisa Maria Fazzi

**Affiliations:** ^1^Division of Anatomy and Physiopathology, Department of Clinical and Experimental Sciences, University of Brescia, Brescia, Italy; ^2^Interdipartimental University Center of Research “Adaption and Regeneration of Tissues and Organs-(ARTO)”, University of Brescia, Brescia, Italy; ^3^Department of Experimental Medicine, University of Campania, Naples, Italy; ^4^European Biomedical Research Institute of Salerno (EBRIS), Salerno, Italy; ^5^Department of Cellular and Integrative Physiology, Center for Biomedical Neuroscience, University of Texas Health San Antonio, San Antonio, TX, United States; ^6^Unit of Clinical Pharmacology, Department of Biomedical and Clinical Sciences L. Sacco, “Luigi Sacco” University Hospital, University of Milan, Milan, Italy; ^7^Faculty of Medicine, University of Tours, Tours, France; ^8^Department of Clinical and Experimental Sciences, University of Brescia, Brescia, Italy; ^9^Unit of Child and Adolescent Neurology and Psychiatry, ASST Spedali Civili di Brescia, Brescia, Italy

**Keywords:** antioxidants, oxidative stress, sleep disturbance, autism spectrum disorders, nutraceutical supplements

Autism spectrum disorders (ASD) represent a heterogeneous group of complex neurodevelopmental disorders associated with atypical behaviors characterized by two core pathological manifestations: deficits in social interaction/communication and repetitive behaviors (1). These impairments vary extensively in symptoms and severity and may go unrecognized when associated with other more disabling conditions. Moreover, some patients with ASD have secondary alterations due to an increase in psychological stress, among them irregular sleep with circadian rhythm alterations, depression, and anxiety (2, 3). The prevalence of ASD has increased over the last several decades, so these disorders are increasingly being recognized as a major public health issue.

Unlike other neurodevelopmental pathologies, the etiology of ASD remains poorly understood; therefore, recently, the number of biological studies of ASD have increased to determine the role of environmental factors in the etiology and pathophysiology of ASD (4).

Furthermore, it was found that oxidative stress and genetic variability in glutathione S-transferases (GSTs) might increase the risk of ASD development. GST polymorphisms may influence the severity of symptoms as well as the cognitive and adaptive functions in children with ASD, showing a complex interaction between genetic and environmental factors in ASD (5).

To date, there are several hypotheses of the mechanism underlying the development of ASD at both cellular and molecular level (6). Multiple factors including an excess of neurons resulting in local hyperconnectivity in key brain regions, unbalanced networks, disturbance in the neuronal migration during early gestation, or abnormal formation of dendritic spines or synapses have been associated with ASD development (7).

Recently several lines of evidence suggest the role of oxidative stress in the pathogenesis of this group of diseases. Indeed, it has been demonstrated that ASD patients are more prone to undergo oxidative stress and are very vulnerable to reactive oxygen species-mediated damage and neuronal toxicity. Oxidative stress occurs when there is an imbalance between the synthesis of reactive oxygen/nitrogen species (ROS/RNS) and the organism's ability to block their deleterious effects using endogenous antioxidative enzymes. In certain neuropsychiatric disorders (including anxiety disorders), attention deficit hyperactivity disorder, and bipolar disorder, this imbalance together with enhanced oxidative stress leads to neuronal damage in genetically predisposed people.

Previous studies also indicate that children with ASD have lower levels of glutathione, an antioxidant molecule. Moreover, a reduction in superoxide dismutase (SOD) activities was reported in autistic children compared to healthy controls. Similarly, vitamin E levels were reported to be consistently lower in ASD children than in healthy controls.

Furthermore, recent ASD studies documented a reduced total antioxidant capacity (TAC), higher levels of 8-hydroxy-2′- deoxyguanosine (8-OHdG, a biomarker of oxidative modifications to DNA), and elevated urinary levels of hexanoyl-lysine (HEL, a new biomarker of oxidative stress). In this regard, Imataka et al. conducted the first study of the role of a set of urinary and plasma oxidative stress-related biomarkers and the association between these urinary and plasma biomarkers and ASD.

They showed that urinary antioxidant biomarker HEL was increased, while urinary biomarker TAC levels were reduced in the ASD group. These data suggest that a critical imbalance between urinary TAC levels and plasma SOD levels may be an important contributor to autistic behavioral symptoms.

Given these findings, oxidative stress may play a crucial role in the etiology of ASD, inducing both structural and functional neuronal damage by different pathways in vulnerable individuals.

Recent research explored supplementation with antioxidant agents as a potential therapeutic/complementary strategy for ASD, while avoiding established pharmacological treatments that may produce significant side effects.

This Research Topic reports some important studies evaluating the effect of alternative natural treatment on behavioral symptoms and most common comorbidities of the disease, including sleep disturbances, while focusing on treatment antioxidant effect.

Zambrelli et al. reviewed 21 papers focusing on the effects of antioxidant agent supplementation on sleep in ASD. More of them, 15 studies, involved melatonin, one studied tryptophan, and the others evaluated supplementation with other antioxidant agents, such as Coenzyme Q10, L-Carnosine, luteolin, and quercetin. The authors concluded that, despite the high prevalence of comorbid sleep troubles in ASD, there is a paucity of data on the efficacy of antioxidant agents in those patients, and that further research is needed to better define the role of antioxidant agents as adjunctive therapy in the management sleep disorders in children and adolescents with ASD.

Galli et al. confirmed that children with ASD showed a high prevalence of sleep disturbances, in particular insomnia, which could be associated with developmental/cognitive delay, emotional and behavior problems, and poor sleep hygiene. The results suggested that regular administration of melatonin, a well-known antioxidant molecule, may ameliorate sleep disturbances.

Moreover, Castejon et al. showed that nutritional supplement comprising a cysteine-rich whey protein isolate (CRWP), a potent precursor of glutathione, improves behaviors and intracellular glutathione levels in 3–5 year old preschool children with confirmed ASD. The authors used a comprehensive behavioral assessment to explore the impact of this supplementation. The results demonstrated that several of the behavioral scales did not show significant differences when comparing changes between the two groups from baseline to follow-up, but demonstrated a favorable effect of this supplement on several aspects of ASD behavior. Moreover, an improvement in antioxidant capacity was demonstrated by increased glutathione levels.

Cucinotta et al., in a retrospective chart review, postulated that metabolic support therapy with Q10 ubiquinol, vitamin E, and complex-B vitamins is well-tolerated and produces some improvement in most patients with neurodevelopmental disorders, especially in the presence of an intellectual disability. The most widespread improvements were recorded in cognition, adaptative functioning, and social motivation. These data provide preliminary evidence that this therapy may represent a safe and viable strategy, which is able to partially ameliorate cognitive, adaptive, social, and motor functions in children, adolescents, and adults in several neurodevelopmental disorders.

Collectively, the articles comprising this Research Topic provide novel insight into adding further knowledge and research opportunities for the development of new nutraceutical/adjunctive strategies for ASD prevention and treatment.

## Author Contributions

All authors listed have made a substantial, direct, and intellectual contribution to the work and approved it for publication.

## Conflict of Interest

The authors declare that the research was conducted in the absence of any commercial or financial relationships that could be construed as a potential conflict of interest.

## Publisher's Note

All claims expressed in this article are solely those of the authors and do not necessarily represent those of their affiliated organizations, or those of the publisher, the editors and the reviewers. Any product that may be evaluated in this article, or claim that may be made by its manufacturer, is not guaranteed or endorsed by the publisher.
